# Transmembrane topology and oligomeric nature of an astrocytic membrane protein, MLC1

**DOI:** 10.1098/rsob.210103

**Published:** 2021-12-01

**Authors:** Junmo Hwang, Kunwoong Park, Ga-Young Lee, Bo Young Yoon, Hyunmin Kim, Sung Hoon Roh, Byoung-Cheol Lee, Kipom Kim, Hyun-Ho Lim

**Affiliations:** ^1^ Neurovascular Unit Research Group, Korea Brain Research Institute (KBRI), 61 Cheomdan-ro, Dong-gu, Daegu 41068, Republic of Korea; ^2^ Brain Research Core Facility, Korea Brain Research Institute (KBRI), Daegu, Republic of Korea; ^3^ School of Biological Science, Institute of Molecular Biology and Genetics, Seoul National University, Seoul, Republic of Korea; ^4^ Department of Brain and Cognitive Sciences, Daegu Gyeongbuk Institute of Science and Technology (DGIST), Daegu, Republic of Korea

**Keywords:** MLC1, membrane protein, transmembrane topology, subunit stoichiometry, fluorescence quenching

## Abstract

MLC1 is a membrane protein mainly expressed in astrocytes, and genetic mutations lead to the development of a leukodystrophy, megalencephalic leukoencephalopathy with subcortical cysts disease. Currently, the biochemical properties of the MLC1 protein are largely unknown. In this study, we aimed to characterize the transmembrane (TM) topology and oligomeric nature of the MLC1 protein. Systematic immunofluorescence staining data revealed that the MLC1 protein has eight TM domains and that both the N- and C-terminus face the cytoplasm. We found that MLC1 can be purified as an oligomer and could form a trimeric complex in both detergent micelles and reconstituted proteoliposomes. Additionally, a single-molecule photobleaching experiment showed that MLC1 protein complexes could consist of three MLC1 monomers in the reconstituted proteoliposomes. These results can provide a basis for both the high-resolution structural determination and functional characterization of the MLC1 protein.

## Graphical abstract

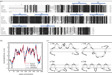

## 1. Introduction

Megalencephalic leukoencephalopathy with subcortical cysts (MLC) is a rare early-onset leukodystrophy caused by genetic mutations in the genes encoding glial membrane proteins MLC1 and GlialCAM. The pathological outcome of MLC disease is impaired motor coordination, cognitive function and epilepsy. The genetic aetiology indicates that approximately 75% of affected patients carry genetic mutations in the *MLC1* gene [1–10]. Previous studies have suggested that MLC1 is crucial for astroglial interactions and water homeostasis, which might cause the pathological outcomes of MLC disease [11–13]. However, the physiological function of MLC1 remains elusive, and the dearth of information about the structure and biochemical properties of the MLC1 protein further limits our understanding of the link between mutations and MLC disease.

The transmembrane (TM) topology of the MLC1 protein was initially predicted to consist of six TM segments based on marginal amino acid sequence homology with a voltage-gated K^+^ channel, KCNA1 [14]. Alternatively, eight TM domains were predicted from the hydropathy plot analysis [15]. However, none of these predictions is supported by experimental data. The only experimentally supported idea regarding the TM topology of MLC1 is that both the amino and carboxylic termini of mouse MLC1 protein reside in the cytoplasmic face [11,15], which suggests that MLC1 protein should contain an even number of TM segments. Although the current bioinformatics tools can reliably generate the TM topology, the primary sequence-based prediction of the TM topology limits the interpretation of MLC1: the number of TM domains in MLC1 can vary from two to eight depending on the prediction algorithms (electronic supplementary material, figure S1).

Both static and dynamic oligomeric states of membrane proteins are critical for conferring specific activities on membrane proteins. For example, static multimeric architectures of ion channels (e.g. dimeric CLC Cl^−^ channels, trimeric ASIC channels, tetrameric K^+^ channels and pentameric ligand-gated ion channels) are crucial for the ligand binding, gating and ion selectivity of channel proteins [16–20]. By contrast, cellular signalling is transduced by the dynamic oligomerization of G-protein-coupled receptors [21–23]. A previous study showed that MLC1 proteins from both mouse brain extracts and transfected HeLa cells are separated into two and three distinct bands in SDS gels, respectively. Additionally, assembly-dependent trafficking assays and split-TEV assays have revealed that MLC1 can form an oligomer *in vivo* [11,24]. These results suggest that MLC1 might be a dimeric membrane protein, but the oligomeric nature of the MLC1 protein in both its purified form and the cell membrane is largely unknown.

Because the TM topology and oligomeric state are important for controlling the biological function of membrane proteins, the quantitative access to these proteins is not a trivial question that can provide a clue to improve the understanding of the molecular action of uncharacterized membrane proteins. In this study, we aimed to determine the TM topology and oligomeric state of the human MLC1 protein. By combining systematic epitope labelling, chemical cross-linking, size-exclusion chromatography (SEC) with multi-angle light scattering-refractive index (SEC-MALS-RI), negative staining electron microscopy (EM) and single-molecule fluorescence quenching, we found that human MLC1 may form a trimeric architecture of eight TM monomeric proteins.

## 2. Results

### 2.1. Primary amino acid sequence conservation of MLC1

**Figure 1.**
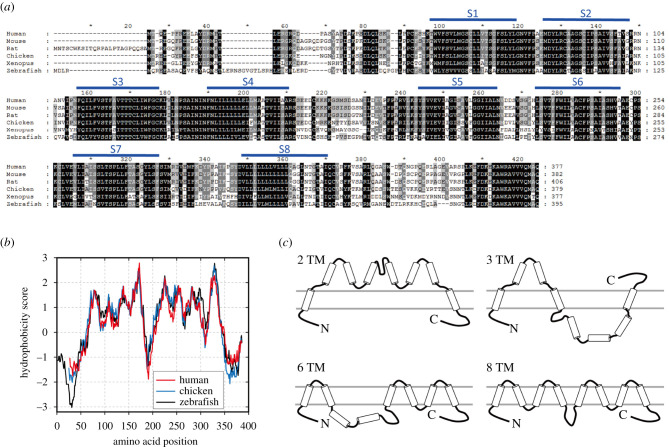
Predicted TM topology of the MLC1 protein. (*a*) Amino acid sequence alignment of MLC1 proteins from vertebrates. The lines indicate the predicted hydrophobic segments based on the human orthologue. (*b*) Hydropathy plot analysis of human (red), chicken (blue) and zebrafish (black) MLC1 proteins. The hydrophobicity of each MLC1 was calculated using the Kyte & Doolittle method in the ProtScale analysis server (web.expasy.org/protscale) [61]. The analysis window size for the calculations was 19 amino acids. (*c*) Cartoon of TM topology models from different prediction algorithms in the TOPCONS web server (https://topcons.cbr.su.se) [25].

The *MLC1* genes are evolutionarily conserved in the phylum Vertebrata (OrthoDB, NCBI gene ID 23209). An amino acid sequence alignment of six MLC1 proteins from Euteleostomi, a clade of bony vertebrates, showed that approximately 50–90% of amino acids are identical among the species (figure 1*a*; electronic supplementary material, figure S1A). Additionally, the amino acid composition and hydrophobicity of putative TM segments are well conserved among the species (figure 1*a,b*), which indicates that the TM topology of MLC1 could also be conserved. However, various numbers (two, three, six and eight) of TM domains in the MLC1 protein have been estimated by TM prediction algorithms (figure 1*c*; electronic supplementary material, figure S1B) [25,26].

### 2.2. Transmembrane topology of hMLC1

**Figure 2.**
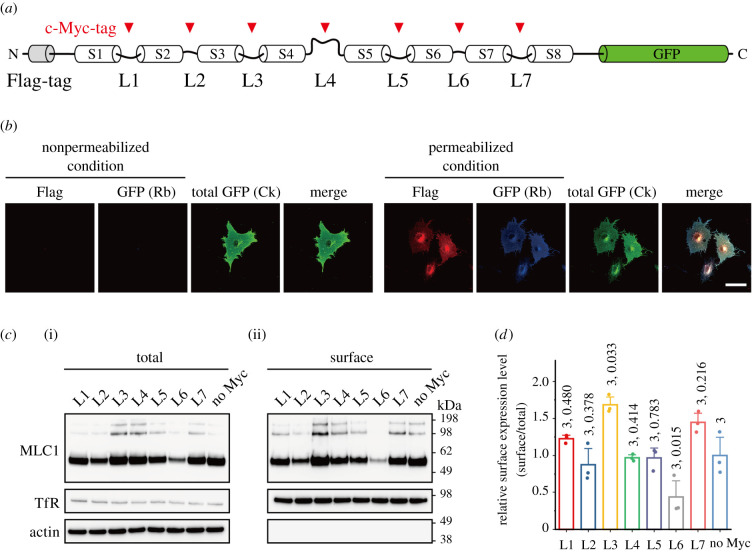
Expression and membrane targeting of the c-Myc-tagged hMLC1. (*a*) Schematic of F-hMLC1-G constructs with c-Myc tags in putative loops. (*b*) Immunofluorescence staining of COS-7 cells expressing F-hMLC1-G. For nonpermeabilized condition, Flag (red) and GFP (blue, with rabbit (Rb) anti-GFP antibody) were stained before permeabilization. To confirm the expression of F-hMLC1-GFP, the C-terminal GFP was visualized with chicken (Ck) anti-GFP antibody (green) after permeabilization. By contrast, for permeabilized condition, three antibodies (anti-Flag, Rb anti-GFP and Ck anti-GFP) were simultaneously stained. Scale bar: 50 µm. (*c*) Total expression level (i) and surface expression level (ii) of various MLC1 constructs. The nomenclature of each construct represents the position of c-Myc tag insertion as in figure 2*a*. (*d*) Relative surface expression level of each protein normalized to the total expression level.

To determine the TM topology of MLC1, we used a human MLC1 (hMLC1) construct tagged with N-terminal Flag and C-terminal GFP (F-hMLC1-G, figure 2*a*). In a previous study, we showed that the expression and membrane targeting of MLC1 were not affected by the N- and C-terminal tags [13]. First, we tested the cellular localization of the N- and C-termini of hMLC1 to determine whether both termini reside in the cytoplasmic face as in mouse MLC1 [27]. COS-7 cells expressing F-hMLC1-G were labelled with anti-Flag and anti-GFP antibodies under either detergent-permeabilized or nonpermeabilized conditions. Both antibodies were able to label COS-7 cells transfected with F-hMLC1-G in detergent-permeabilized conditions but not in nonpermeabilized conditions (figure 2*b*). These results indicated that both N-terminal Flag and C-terminal GFP tags on human MLC1 reside intracellularly and confirmed the previous result showing that both the N- and C-termini of mouse MLC1 localize in the cytoplasmic face [27].

**Figure 3.**
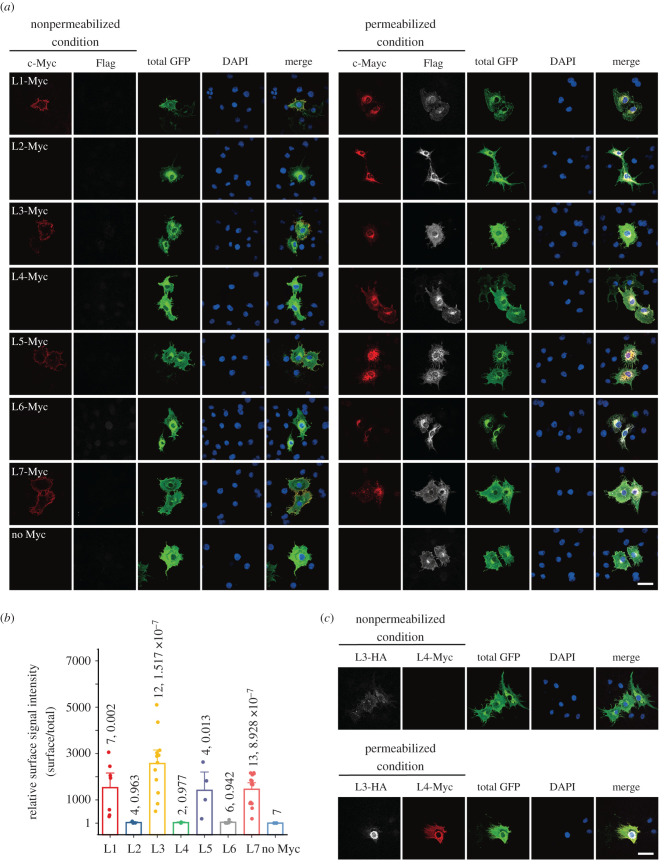
Determination of the TM topology of hMLC1. (*a*) Immunofluorescence staining of COS-7 cells expressing L1-Myc to L7-Myc under either permeabilized or nonpermeabilized conditions. c-Myc (red) and Flag (grey) antibodies were treated before permeabilization to prevent staining of intracellular MLC1 in the nonpermeabilized condition. GFP (green) antibody was treated after permeabilization to use as a positive control for MLC1 in both conditions. The N-terminus of MLC1 (facing cytoplasm) was stained with Flag antibody and used as a negative control for nonpermeabilized condition. In the permeabilized condition, all antibodies were treated after permeabilization. Scale bar: 50 µm. (*b*) Relative surface signal intensity normalized to the total signal intensity. From the confocal images which were stained as figure 2*b*, ROI was determined to isolate a single cell and signal intensity was analysed. c-Myc signal intensity (surface hMLC1) was divided by GFP signal intensity (total hMLC1). One-way ANOVA analysis was performed and *p*-values between No Myc and other constructs were calculated by Fisher's test using Origin 10.6 (OriginLab). *n*- and *p-*values are indicated above each bar. Data are represented as a mean ± s.e. (*c*) Immunofluorescence staining of COS-7 cells expressing hMLC1 which is double-tagged at L3 and L4 regions with HA and Myc, respectively. Scale bar: 50 µm. HA (grey) and c-Myc (red) antibodies were treated as in figure 3*a*.

The TM topology of hMLC1 was then determined using F-hMLC1-G with a c-Myc tag in the putative loops (L1–L7) between the predicted eight TM domains (figure 2*a*; electronic supplementary material, figure S2). To confirm that all these c-Myc-tagged MLC1 proteins can reach the plasma membrane, cell surface biotinylation experiment was conducted (figure 2*c*). The surface expression level of c-Myc-tagged MLC1 proteins normalized to the total expression level showed that surface localization is comparable to each other (figure 2*d*). Though the relative surface expression level of L6-Myc is about half of those of other proteins, it should not affect the sensitivity for discriminating detergent-permeabilized and nonpermeabilized staining patterns. Then, the antibody accessibility to the c-Myc tag on either the cytoplasmic or extracellular side was tested by comparing the immunofluorescence staining patterns between detergent-permeabilized and nonpermeabilized conditions as described above. For example, a c-Myc tag placed in putative loop 1 between TM domains 1 and 2 (L1-Myc) was stained under both permeabilized and nonpermeabilized conditions, but L2-Myc was stained only under permeabilized conditions (figure 3*a*). These alternating staining patterns indicated that L1 was exposed to the cell surface and L2 was placed in the cytoplasm. Thus, systematic c-Myc staining revealed that four out of seven putative loops (L1, L3, L5 and L7) are exposed to the extracellular side, whereas the other three (L2, L4 and L6) reside in the cytoplasmic face (figure 3*a*). The surface intensity (c-Myc signal intensity) normalized to the total signal intensity (total GFP signal intensity) clearly indicates that MLC1 has eight TM domains (figure 3*b*). To validate the results from the antibody accessibility tests, the immunofluorescence staining patterns of an F-hMLC1-G with an HA-tag in the L3 position and c-Myc in the L4 position (L3-HA/L4-Myc) were examined: the HA antibody labelled the cells under both permeabilized and nonpermeabilized conditions, but c-Myc antibody could stain cells only under permeabilized conditions (figure 3*c*). These data suggested that hMLC1 has eight TM segments with both N- and C-termini inside.

An algorithm of the protein structure prediction based on a machine learning, AlphaFold can produce highly accurate three-dimensional (3D) protein structure prediction. And AlphaFold protein structure database was recently open to the public [28]. Predicted 3D structures of MLC1 proteins from human, rat, mouse and zebrafish can be found in the database. AlphaFold structure prediction of human MLC1 shows that eight long alpha helices (h1–h8, 24–45 amino acids long) and a short alpha helix (hct, 13 amino acids long) are parallel to each other (electronic supplementary material, figure S3). Since the median length of TM helices in 235 high-resolution structures of integral membrane protein is 24.9 ± 7.0 residues and the most common length is 23 residues [29], long alpha helices (h1–h8) are enough to pass the membrane, but a short alpha helix (hct) is not likely to the TM helix. And structural comparison among MLC1 orthologues from human, mouse and zebrafish shows that these alpha helices are well conserved (electronic supplementary material, figure S3B). These structural predictions of MLC1 proteins could support our experimental evidence that MLC1 protein has eight TM segments.

### 2.3. Purification and chemical cross-linking MLC1 proteins

**Figure 4.**
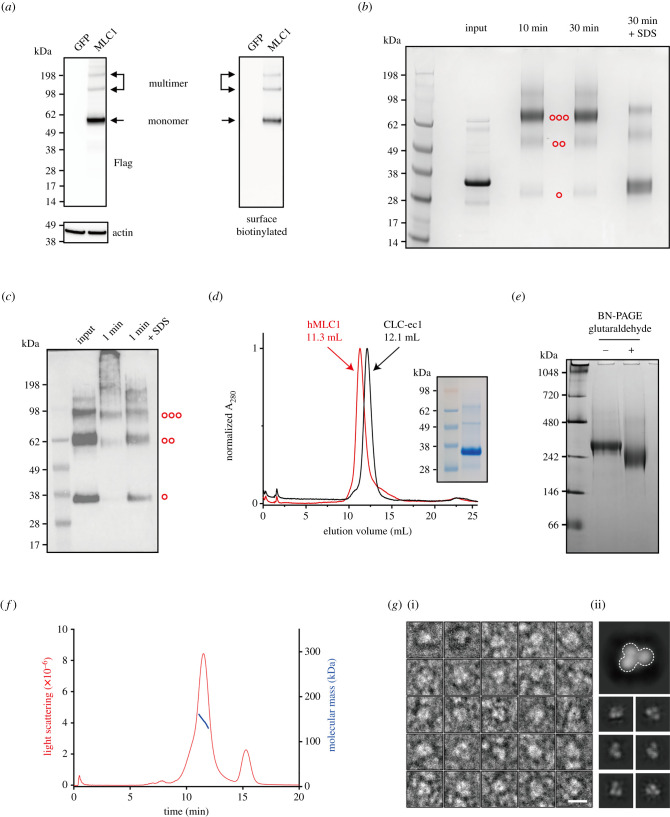
Oligomeric nature of hMLC1 in detergent micelles and reconstituted proteoliposomes. (*a*) Western blot of total and surface (the plasma membrane) F-hMLC1-G protein expressed in COS-7 cells with anti-Flag antibody. (*b*) Cross-linking of purified hMLC1 was performed in detergent micelles and analysed by SDS–PAGE. Glutaraldehyde treatment with or without 2% SDS was performed for 10 or 30 min. (*c*) Glutaraldehyde cross-linking of hMLC1 in the reconstituted liposomes. hMLC1 bands were visualized by western blot analysis with an anti-Flag antibody. (*d*) SEC profile of purified hMLC1 and CLC-ec1. The purity of purified 3× Flag-hMLC1 was verified by SDS–PAGE (inset). (*e*) Blue native (BN)-PAGE of purified 3× Flag-hMLC1 with or without glutaraldehyde cross-linking. (*f*) FPLC-MALS-RI profile of purified hMLC1. The red line indicates light scattering, and the blue line is the estimated molecular mass. (*g*) Negative-EM images of hMLC1. Twenty-five sample images of particles picked (i) and a representative 2D class of trimeric hMLC1 complex (ii). Scale bar: 10 nm.

A previous study indicated that mouse MLC1 ran on the SDS–PAGE in both monomeric and oligomeric forms even in the presence of the ionic detergent SDS [11]. Similarly, three distinct bands for the F-hMLC1-G were detected by western blotting with anti-Flag antibody both in the transfected total cell lysate and in the surface-biotinylated sample in SDS-PAGE. The band sizes were estimated to be approximately 56 kDa, approximately 105 kDa and approximately 189 kDa, respectively. (figure 4*a*). It should be noted that the F-hMLC1-G protein moved faster than the predicted molecular weight of approximately 69 kDa in the SDS–PAGE, as described in the previous reports [30,31]. This anomalous migration of MLC1 could be due to detergent-binding to the helical membrane protein in SDS–PAGE [32]. The multimeric bands of MLC1 in the SDS–PAGE suggest that MLC1 might be present in an oligomeric state, but other factors, such as glycosylation, phosphorylation, SUMOylation, ubiquitination and interactions with binding molecules, can draw an improper conclusion.

To rule out the possibility of multiple protein bands in the SDS–PAGE other than the oligomerization of MLC1, we aimed to examine the oligomeric nature of MLC1 protein purified from cells. To do this, the hMLC1 tagged with N-terminal 3× Flag and codon-optimized ckMLC1 (chicken MLC1) tagged with a C-terminal 6× His-tag were expressed in HEK293S GnTI^−^ cells and in *E. coli* BL21(DE3) cells, respectively, and purified by using affinity chromatography and SEC. The oligomeric state of purified 3× Flag-tagged MLC1 proteins was assessed by chemical cross-linking with glutaraldehyde, which has been used to examine the oligomeric state of various membrane proteins [30,31,33,34]. Glutaraldehyde treatment shifts hMLC1 from the monomeric position (approx. 30.2 kDa) to oligomerized positions (estimated sizes of approx. 53.2 and approx. 74.1 kDa in SDS-PAGE). The major cross-linking products were found in the trimeric position, which suggests that it may exist in the trimeric state in detergent micelles. By contrast, the cross-linking of hMLC1 was largely inhibited in denaturing condition with SDS, which could interfere with subunit interactions in solution (figure 4*b*). Additionally, the results from the glutaraldehyde cross-linking of ckMLC1 support the idea that MLC1 is a homo-trimeric protein (electronic supplementary material, figure S4A). However, the oligomeric state of MLC1 protein in detergent micelle might not represent the native oligomeric state in a membrane environment. Thus, we tested whether MLC1 proteins retain an oligomeric conformation in a lipid environment. To reconstitute purified MLC1 proteins into the phospholipid bilayer, we used the well-established polystyrene beads-mediated reconstitution method [35]. The MLC1 proteoliposomes were treated with glutaraldehyde as above and the reaction samples were separated in SDS-PAGE as in the previous studies [30,31,33,34]. A significant fraction of hMLC1, as well as ckMLC1, was cross-linked in the lipid bilayer (figure 4*c*; electronic supplementary material, figure S4B), which recapitulated the notion of cross-linking results in detergent micelles.

### 2.4. Quantification of MLC1 subunit

Interestingly, the SEC profiles of the purified MLC1 proteins (3× Flag-tagged hMLC1 and 6× His-tagged ckMLC1) showed that both MLC1 proteins move faster (approx. 0.8 ml for hMLC1 and approximately 0.6 ml for ckMLC1) than the control membrane protein CLC-ec1 dimer, which has a known molecular weight of approximately 100 kDa [30] (figure 4*d*; electronic supplementary material, figure S4C). These results indicate that MLC1 could migrate as a larger molecular complex than CLC-ec1 dimer in the solution with detergent micelle. Also, the purified 3× Flag-tagged hMLC1 migrated as a single distinct band of approximately 250 kDa in a blue native PAGE (BN-PAGE) in the absence and presence of a chemical cross-linker, glutaraldehyde (figure 4*e*). It suggests that MLC1 could form a stable oligomeric complex in the purified form as well as the glutaraldehyde cross-linking did not produce an artificial oligomeric state of MLC1 protein. However, both SEC and BN-PAGE are unsuitable to estimate the molecular weight of membrane proteins complexed with the unknown number of detergent molecules.

Thus, we tried to estimate molecular weight of purified MLC1 protein complex by using the SEC coupled with multi-angle light scattering and differential refractive index (SEC-MALS-RI) system to obtain a quantitative idea of the MLC1 subunit stoichiometry. The SEC-MALS-RI system, which enables to measure the number of detergent molecules in the membrane protein-detergent micelle, has been successfully used to report the molecular weight of several membrane proteins in detergent micelle by a simultaneous measure of UV, MALS and RI [36–38]. As the internal control, proteins with known molecular weight and oligomeric assembly (bovine serum albumin, approximately 66 kDa as a monomer; CLC-ec1, approximately 100 kDa as a dimer; and CLC-ck2, approximately 89 kDa as a dimer [39]) were examined by using the SEC-MALS-RI. The estimated sizes from SEC-MALS-RI were approximately 73 kDa, approximately 107 kDa and approximately 100 kDa, respectively (electronic supplementary material, figure S5). These estimated values for control proteins are only 7–12% off from the predicted molecular weights, which indicated that the SEC-MALS-RI system can reliably estimate the molecular weight of both membrane and soluble proteins. And purified 3× Flag-tagged hMLC1 (predicted molecular weight of monomer, approximately 44 kDa) was subjected to the SEC-MALS-RI. The estimated molecular weight of hMLC1 in DDM detergent measured by SEC-MALS-RI was approximately 146 kDa (figure 4*f*). This estimated size of hMLC1 complex is close to the predicted molecular weight of the 3× Flag-tagged hMLC1 trimer (approx. 132 kDa). To visualize the trimeric architecture of MLC1 protein, we performed negative staining EM. We picked 43 193 particles from 98 negative-EM micrographs. Two-dimensional (2D) classification of 17 133 particles from template-based picking shows that the dominant feature of hMLC1 is supposed to be a trimeric complex (figure 4*g*). These results from the quantitative approaches strongly suggested that the purified hMLC1 in detergent micelles forms a homo-trimeric complex.

### 2.5. Subunit stoichiometry of MLC1 in the membrane environment

**Figure 5.**
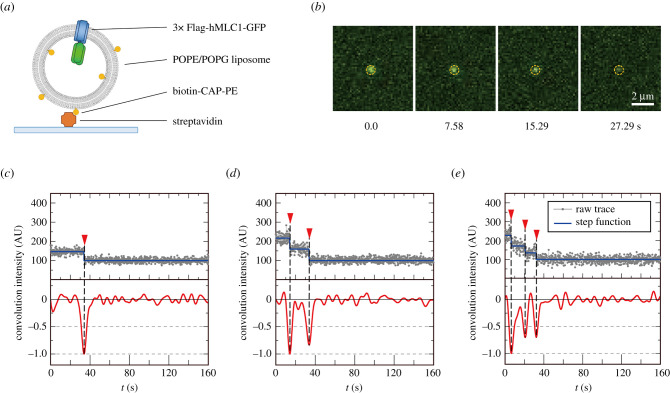
Photobleaching of F-hMLC1-G reconstituted proteoliposomes showed multiple bleaching steps. (*a*) Cartoon depicting the single-molecule photobleaching imaging of reconstituted 3× F-hMLC1-G in the proteoliposomes immobilized on the streptavidin-coated coverslip. (*b*) A representative snapshot of bleaching particle at the indicated time. (*c–e*) Examples of fluorescence intensity trace with distinct photobleaching events. The blue graph above raw traces (grey) represents the mean value between two nearby bleaching events. The red graph is the convolution with derivative-of-Gaussian wavelets to indicate the peaks representing stepwise bleaching events. The red arrowheads present stepwise decrease events in the trace.

Single-molecule photobleaching has become a popular tool to examine the subunit stoichiometry of protein complexes. By attaching fluorophores (typically GFP or a variant) to subunits of interest, the number of the labelled subunits in a complex can be assumed by single-molecule imaging and counting fluorophore photobleaching steps. During sufficient excitation, a fluorophore will bleach, resulting in a stepwise decrease in observed fluorescence intensity. Then, the number of labelled subunits in the complex can be analysed by counting these bleaching steps. To quantify the subunit stoichiometry of MLC1 protein in the membrane environment, we examined the single-molecule photobleaching of 3× Flag-tagged hMLC1-GFP in the reconstituted proteoliposomes with total internal reflection fluorescence microscopy (TIRFM) (figure 5). As in the previous studies, protein was reconstituted at low densities (0.4 mg protein per mg lipid) to minimize over-filing of hMLC1 subunit [40–42]. To obtain the photobleaching patterns of the 3× Flag-tagged hMLC-GFP proteoliposomes, the biotin-CAP-PE containing proteoliposomes were immobilized onto the streptavidin-coated coverslip (figure 5*a*). The images were acquired until most fluorophores were fully bleached and the complexes were then identified in the initial frame of the time-series image, and the fluorescence intensity of each complex was tracked in subsequent frames. To analyse the photobleaching events of the regions of interest (ROIs), we chose the GFP signals which were well separated from the others (figure 5*b*) and showed well-distinguishable bleaching events were selected, and the number of bleaching events was counted from the intensity traces. To make unbiased step counting, the intensity traces were convoluted with the first derivative-of-Gaussian wavelets, and then positions of the deep valleys below –0.5 were recognized as photobleaching event locations [43,44] (figure 5*c–e*, red graphs). Bleaching events were visualized as a step with a horizontal mean value line between each step (figure 5*c–e*, blue lines). The fluorescence intensities decreased in a stepwise manner, which corresponds to the turn-off event of GFP tethered to each MLC1 subunit in the complex (figure 5*c–e*, *red* arrows and dashed black vertical lines).

**Figure 6.**
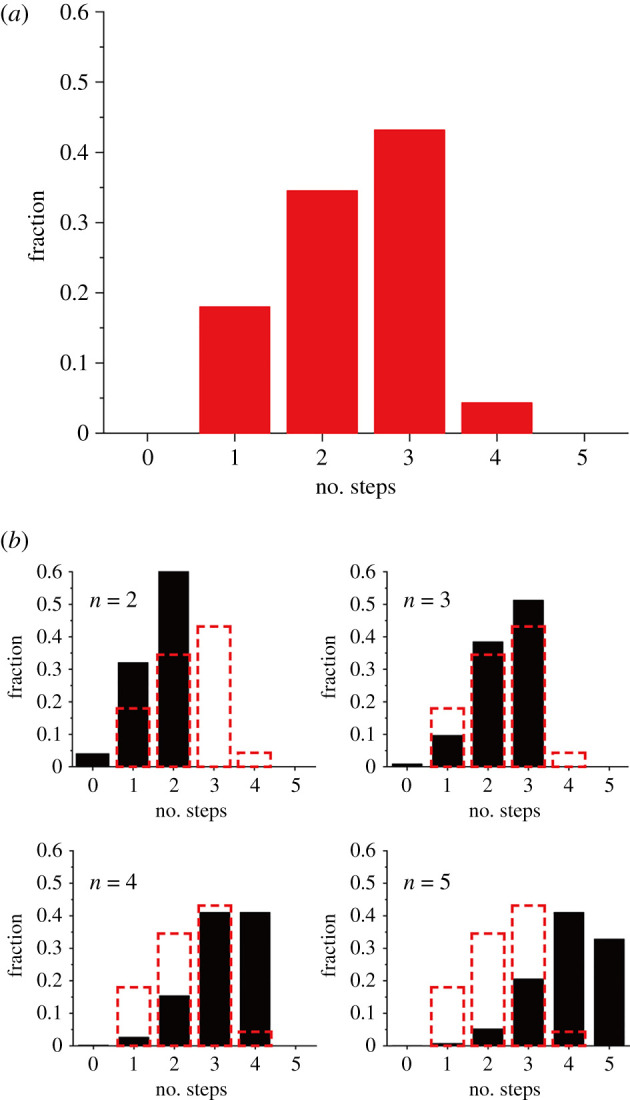
Analysis of photobleaching steps of F-hMLC1-G proteoliposomes. (*a*) Distribution of photobleaching events observed in 3× F-hMLC1-GFP proteoliposomes. (*b*) the binomial distribution models of various subunit models (black bar graphs). The ‘*n*’ indicates subunit numbers in each model with a probability of 80% of the GFP to be fluorescent (equation (4.1) in Material and methods). The dashed red bar graph indicates the observed photobleaching distribution in (*a*).

To estimate the subunit stoichiometry of 3× Flag-tagged hMLC1-GFP reconstituted in the proteoliposomes, the photobleaching event distribution was plotted (figure 6*a*) and compared with the binomial distribution with various subunit models (figure 6*b*). Since the GFP protein is fused to the C-terminus of hMLC1, one may expect that the fluorescence detection probability of the subunit should be closed to 100%. However, the photobleaching of the various membrane proteins (calcium channel, NMDA receptor, CNG channel, and BEST channels) tagged with the GFP showed that approximately 80% of the GFPs are fluorescent at the start of TIRF imaging, which may be due to misfolding or incomplete maturation of the GFP [45,46]. Thus, we applied a probability of 80% (*p* = 0.8) of the GFP to be fluorescent to calculate the binomial distribution models (figure 6*b*, equation (4.1) in Material and methods). And the goodness of the models was evaluated by the sum of squares (SS) of models (equation (4.2) in Material and methods). The SS values are 0.296 for dimer, 0.018 for trimer, 0.189 for tetramer and 0.401 for pentamer, respectively. Thus, the measured distribution was the best matched with the binomial distribution assuming trimeric complex. The single-molecule photobleaching data present here suggest that the hMLC1 complex is most likely to form a trimeric architecture in the membrane environment.

## 3. Discussion

Mutations in the MLC1 protein can cause a neurological disorder named MLC disease. More than 50 *MLC1* mutations, including splice-site, nonsense, missense, deletion and insertion mutations, have been reported [47–50], and 35 of these mutations are caused by single amino acid substitutions. Interestingly, 27 of these mutations were in the predicted TM domains, and four were closely adjacent to the TM domains (electronic supplementary material, figure S2, red and magenta letters, respectively). Previous studies showed that some MLC1 missense mutations are trapped in intracellular compartments in heterologous expression systems, such as *Xenopus* oocytes, HeLa cells and COS-7 cells [13,27]. These experimental observations suggest that missense mutations in the TM helices might disrupt the proper folding or oligomerization needed for functional targeting of the MLC1 protein. It will be interesting to test whether these mutant proteins maintain proper folding and oligomerization, as described here for wild-type MLC1.

The TM topology data showed that MLC1 has three loops (L2, L4 and L6) exposed to the cytoplasmic side between TM domains (figure 3*a*; electronic supplementary material, figure S2). The structural models of MLC1 orthologues from AlphaFold prediction showed that MLC1 proteins have conserved eight TM domains packed parallel to each other. These predictions support our experimental data, in which MLC1 has eight TM domains. Among the loops between TM domains, L4 (from Arg-165 to Ser-197) is relatively longer than the other loops. Interestingly, heavily clustered serine residues (Ser-166, Ser-167, Ser-177, Ser-179, Ser-181 and Ser-197) were found in L4. Previous studies discovered that Ser-27 and Ser-339 of MLC1 is the phosphorylation sites for PKA/PKC, which were related to plasma membrane trafficking [51]. The presence of additional central loop-condensed serine residues implies that MLC1 might be regulated by cooperative phosphorylation, as observed in the phosphorylation of cyclin B1 by MPF, MAPK and Plk1 kinases [52].

Interestingly, Raúl Estévez's group performed a series of biochemical studies, including assembly dependent trafficking assays and split-TEV assays, and found that MLC1 proteins can exist in a homo-oligomeric form [11,24]. These results suggest that the MLC1 protein might assemble into a homodimeric architecture with an antiparallel structure in which the N-terminal domain interacts with the C-terminal part of the neighbouring subunit [53]. However, these results cannot rule out the possibility of higher oligomeric states. Together, our data were obtained using multiple experimental approaches, including SEC, chemical cross-linking, SEC-MALS-RI and negative staining EM with MLC1 proteins in either purified detergent micelles or reconstituted bilayer membranes, as well as the single-molecule photobleaching of MLC1 in the reconstituted proteoliposomes and in the cell membrane suggest that MLC1 could present as a homo-trimeric complex. However, the homo-trimeric assembly of MLC1 needs to be interpreted with caution since the experimental approaches presented here ruled out the effect of a known MLC1-interacting protein, GlialCAM. It has been suggested that an MLC1-interacting protein, GlialCAM, is present as a dimer in the plasma membrane [54], and its mutations can cause MLC disease [3,55]. Thus, it will be interesting to test whether wild-type and mutant GlialCAM can modify the assembly of the MLC1 complex.

Single-molecule photobleaching experiments have been widely used to elucidate the subunit stoichiometry of various membrane proteins [40,56,57]. However, the results should be interpreted with caution. For example, single-molecule imaging has led to an incorrect conclusion regarding the subunit stoichiometry of the Bestrophin channels [45]. Thus, a high-resolution structure of MLC1 is needed to draw a conclusion regarding the oligomeric states of the MLC1 protein. Recent progress in membrane protein structural biology, including cryo-EM, can unveil the oligomeric state of MLC1 with atomic resolution in the near future.

In summary, we characterized the astrocytic membrane protein MLC1 at the protein level. Through systematic immunofluorescence staining of the epitope tag on the loops between putative TM domains, we revealed that hMLC1 has eight TM segments with both the amino- and carboxyl-termini exposed to the cytoplasmic face. Moreover, we found that hMLC1 proteins were trimeric in an isolated protein-detergent micelle and a reconstituted bilayer membrane. These results may provide a framework for determining high-resolution structures at the atomic level and for the functional characterization of wild-type and pathogenic mutant MLC1 proteins.

## 4. Material and methods

### 4.1. Reagents

n-Dodecyl-β-D-maltopyranoside (DDM), 2,2-didecylpropane-1,3-bis-β-D-maltopyranoside (lauryl maltose neopentyl glycol [LMNG], NG310) and CHAPS were purchased from Anatrace (Maumee, OH). Poloxamer (P5556), anti-Flag M2 affinity gel (A2220, RRID:AB_10063035), 3× Flag peptide (F4799), glutaraldehyde (G5882), Poloxamer 188 solution (10%, P5556) and anti-Flag (F1804, RRID:AB_262044) antibody were purchased from Sigma-Aldrich (St Louis, MO). PFA (15710) was purchased from Electron Microscopy Sciences (Hatfield, PA). COS-7 (CRL-1651) and HEK293S GnTI^−^ cells (CRL-3022) were purchased from ATCC (Manassas, VA). Fetal bovine serum (FBS, 16000-044), Dulbecco's modified Eagle's medium (DMEM, 11995073), penicillin–streptomycin (15140122), Opti-MEM (31985-062), FreeStyle 293 Expression Medium (12338018), ProLong Gold antifade solution (P36981) and anti-GFP (A10262 (RRID:AB_2534023) and A11122 (RRID:AB_221569)) antibodies were purchased from Thermo Fisher Scientific (Waltham, MA). Transporter 5 Transfection Reagent (26008) was purchased from PolyScience (Warrington, PA). Anti-Myc (2278S, RRID:AB_490778) and anti-actin (8457S, RRID:AB_10950489) were purchased from Cell Signalling (Danvers, MA). L-α-phosphatidylcholine (EggPC, 840051C), 1-palmitoyl-2-oleoyl-sn-glycero-3-phosphoethanolamine (16 : 0–18 : 1 POPE, 850757C), 1,2-dipalmitoyl-sn-glycero-3-phosphoethanolamine-N-(cap biotinyl) (16 : 0 Biotinyl Cap PE, 870277) and 1-palmitoyl-2-oleoyl-sn-glycero-3-phospho-(1'-rac-glycerol) (16 : 0–18 : 1 POPG, 840457C) were purchased from Avanti Polar Lipid (Alabaster, AL). Bio-Beads SM-2 Adsorbent (1523920) was purchased from Bio-Rad (Hercules, CA). Endotoxin-free DNA maxi kit was purchased from MACHEREY-NAGEL (Düren, Germany). mPEG-Succinimidyl Valerate (MPEG-SVA-5000) and Biotin-PEG-SVA (Biotin-PEG-SVA-5000) were purchased from Laysan Bio (Arab, AL). TALON metal affinity resin (635504) was purchased from TaKaRa bio (Japan); 400 nm Whatman Nuclepore Track-Etched Membranes (10417104) and columns for SEC were purchased from Cytiva (Marlborough, MA).

### 4.2. Cell cultures

The COS-7 cell line was maintained as recommended. Briefly, subculture was performed every 2 to 3 days with DMEM supplemented with 10% FBS and 100 U ml^−1^ penicillin–streptomycin and kept at 37°C in a 5% CO_2_ incubator. The HEK293S GnTI^−^ cell line was maintained in FreeStyle 293 Expression Medium supplemented with 2% (v/v) FBS and 0.025% (v/v) Poloxamer and grown in a CO_2_ incubator (37°C, 5% CO_2_, humidified) with shaking (150 r.p.m.).

### 4.3. Plasmid construction and transfection

For immunofluorescence staining, human (NM_015166.3) MLC1 was obtained from GenScript and subcloned into pCAG vector in frame with N-terminal Flag and C-terminal GFP. The c-Myc tag (N-EQKLISEEDL-C) was inserted into the putative loops by PCR and sequences were confirmed through the conventional sequencing method. The COS-7 cells were seeded on a PLL-coated coverslip which was placed in a 12-well plate; 10 ng ul^−1^ of DNA was prepared in Opti-MEM and mixed with 60 ng ul^−1^ Transporter 5 transfection reagent. After incubating for 20 min at 23°C, 50 μl of transfection mixture per mL media was used. For protein purification, 3× Flag (N-DYKDHDGDYKDHDIDYKDDDDK-C) was tagged at the N-terminus of hMLC1. 1 × 10^6^ HEK293S GnTI^−^ cells per mL media were prepared for transfection.

### 4.4. Immunofluorescence staining

For image-based determination of the membrane topology, immunofluorescence staining and confocal imaging were performed as described previously [13]. COS-7 cells seeded on poly-L-lysine (PLL)-coated coverslips were transfected with F-hMLC1-G and fixed with a solution containing 4% (w/v) paraformaldehyde (PFA), 1× PBS, pH 7.4 and 0.04 g/mL sucrose for 8 min. The fixed samples were divided into two groups for exposure to permeabilized and nonpermeabilized conditions. For exposure to the permeabilized conditions, cells were incubated with permeabilization solution (0.2% (v/v) Triton X-100 and 1× PBS, pH 7.4) for 10 min and then with blocking solution (3% (w/v) BSA, 0.1% (v/v) Tween-20 and 1× PBS, pH 7.4) for 1 h. Primary antibodies (Flag, c-Myc, and GFP) prepared in blocking solution (2–10 ng µl^−1^) were added for 1 h. The cells were then washed three times with washing solution (0.1% (v/v) Tween-20 and 1× PBS, pH 7.4) and incubated with secondary antibodies. For the unpermeabilized conditions, the solutions for blocking, treating primary antibodies (Flag and c-Myc) and washing were prepared without Tween-20 and treated prior to permeabilization. After the staining of F-hMLC1-G on the extracellular side, the cells were blocked for GFP staining. The cells were incubated with secondary antibodies prepared in blocking solution for 45 min. After washing, coverslips were mounted on glass slides using ProLong Gold antifade solution and cured overnight. Confocal images were obtained using an inverted Nikon ECLIPSE Ti-E confocal microscope equipped with an oil-immersion objective lens (Nikon plan Apochromat 60×/NA 1.40). The antibody reactions and microscopic imaging were performed at 23°C.

### 4.5. Expression and purification of hMLC1 and ckMLC1

3× Flag-tagged hMLC1 was inserted into the pEEV-CAG vector and transfected into HEK293S GnTI^−^ cell line. After 48 h, cells were harvested, resuspended in the lysis buffer (20 mM LMNG, 20 mM HEPES, 150 mM NaCl, 1× protease inhibitor cocktail, pH 7.5) and disrupted by using Dounce homogenizer. Membrane proteins were extracted for 2 h at 4°C with gentle agitation. After the removal of cell debris by centrifugation, the cell lysate was loaded onto a pre-equilibrated anti-Flag M2 affinity gel and washed with wash buffer (0.1 mM LMNG, 20 mM HEPES and 150 mM NaCl, pH 7.5). 3× Flag-hMLC1 was eluted with elution buffer (0.1 mM 3× Flag peptide, 0.1 mM LMNG, 20 mM HEPES and 150 mM NaCl, pH 7.5) and run on a size-exclusion column (Superdex 200) equilibrated with FPLC buffer (0.05 mM LMNG, 20 mM HEPES and 150 mM NaCl, pH 7.5). A fraction containing 3× Flag-hMLC1 was collected and prepared for further assay.

Codon-optimized chicken MLC1 (ckMLC1) for expression in *E. coli* was obtained from GenScript and cloned into the pASK90 vector with an N-terminal hexa-histidine tag. ckMLC1 proteins were expressed and purified from BL21(DE3) cells as described previously [58]. Briefly, *E. coli* cells expressing ckMLC1 were lysed by sonication in 100 mM NaCl and 50 mM Tris-Cl (pH 7.5), and membrane proteins were extracted with 2% (w/v) DDM for 2 h at 4°C. After the removal of cell debris by centrifugation, the cell lysate was first subjected to affinity purification on a cobalt column in CB buffer (100 mM NaCl, 20 mM Tris-Cl, pH 7.5 and 5 mM DDM), and ckMLC1 proteins were eluted with 400 mM imidazole in CB buffer. The proteins were further purified by SEC (Superdex 200) equilibrated with 150 mM NaCl, 20 mM HEPES, pH 7.5 and 5 mM DDM.

### 4.6. Proteoliposome reconstitution

For the reconstitution, a 3 : 1 (w : w) mixture of phospholipids (EggPC : POPG for chemical cross-linking or POPE : POPG for single-molecule imaging) dissolved in chloroform were dried under a N_2_ stream, washed with pantane and dried again. The dried lipid mixture was solubilized by sonication in reconstitution buffer (20 mg lipid mg^−1^ in 150 mM NaCl, 50 mM Na-phosphate, pH 7.0 and 35 mM CHAPS). In photobleaching experiment, 0.1 mol% biotinyl CAP-PE was added to the lipid mixture when dry the lipids. To minimize over-filing of hMLC1 subunit in liposomes, proteins were reconstituted at low densities both in glutaraldehyde cross-linking and in single-molecule photobleaching experiments (0.1–1 µg protein mg^−1^ lipid) [40–42]. The detergents were removed by using the polystyrene bead, Bio-beads SM-2 Adsorbent (8 mg bead mg^−1^ lipid) with four bead exchange steps as described previously [59]. In each step, the protein-lipid mixture was incubated with freshly prepared Bio-beads equilibrated with reconstitution buffer (150 mM NaCl, 50 mM Na-phosphate, pH 7.0) for 2 h, 2 h, 15 h and 2 h, respectively. The resulting proteoliposomes were fast frozen in liquid N2 and stored at −80°C. Before the cross-linking reaction or single-molecule photobleaching experiment, the unilamellar proteoliposomes were prepared by three freeze–thaw cycles followed by extrusion 21 times through a 400 nm pore Track-Etch membrane [60].

### 4.7. Protein cross-linking

A 400-fold molar excess of glutaraldehyde was added to protein-detergent micelles (0.5–0.9 µg protein µl^−1^) or proteoliposomes (0.1–1 µg protein mg^−1^ lipid) for the cross-linking of chicken or human MLC1. To inhibit cross-linking, 2% (w/v) SDS was added. After incubation for 1, 10 or 30 min at 23°C, the reaction was terminated by the addition of 150 mM Tris (pH 8.8).

### 4.8. Size-exclusion chromatography with multi-angle light scattering-refractive index

The molecular weight of the hMLC1 oligomer was determined using a Superdex-200 Increase 10*/*300 GL column coupled with a miniDAWN light scattering detector and an Optilab refractive index detector (Wyatt Technology). Purified hMLC1 proteins at a concentration of 1 mg ml^−1^ in 200 µl were loaded onto the column equilibrated with 150 mM NaCl, 20 mM HEPES, pH 7.5 and 2 mM DDM. Bovine serum albumin, CLC-ec1 and CLC-ec2 were used as the molecular weight standards. The data were analysed using ASTRA 8 software (Wyatt Technology).

### 4.9. Negative staining electron microscopy

Purified 3× Flag-tagged hMLC1 protein sample (approx. 0.015 mg ml^−1^) was negatively stained with 2% of Uranyl Acetate on glow-discharged carbon film grids (Agar Scientific). Samples on EM grids were imaged by using Tecnai T12 microscope (120 kV), with a pixel size of 1.65 Å. Collected images were further processed using cryoSPARC suite (v. 3.1.0). Particles for 2D classification were picked based on templates generated by automated particle picking.

### 4.10. Single-molecule imaging and analysis

For single-molecule imaging of the reconstituted proteoliposomes, fluidic imaging chambers were assembled using a pair of PEGylated slide and coverslip, where double-sided sticky tape (3 M) and Epoxy glue were used for a spacer and sealing. For surface PEGylation, the surface of the slide and the coverslip was functionalized with an amine group via the amino-silanization reaction. Then, the amine-functionalized surfaces were passivated by conjugating NHS-ester polyethylene glycol (PEG), where 1 mg µl^−1^ PEG mixture (mPEG : biotin-PEG = 40 : 1) prepared in 0.1 M sodium bicarbonate (pH 8.5) was used to immobilize the biotinylated proteoliposomes on the coverslips. Just before loading the biotinylated proteoliposomes, 50 µl of 0.1 mg ml^−1^ of Neutravidin solution in T50 buffer (10 mM Tris-HCl, 50 mM NaCl, pH 8.0) was introduced through the inlet hole of the imaging chamber. After 20 min of incubation, the imaging chamber was flushed with 100 µl T50 buffer; 50 µl biotinylated proteoliposomes (0.4 µg 3× Flag-hMLC1-GFP protein mg^−1^ lipid) was introduced and incubated for 20 min. After flushing the imaging chamber with 100 µl PBS buffer, TIRF imaging was conducted. For the membrane-specific imaging and bleaching of F-hMLC1-G, a TIRF microscope was used. TIRF microscopy imaging was performed with a Nikon Ti-E inverted fluorescence microscope (at Brain Research Core Facilities, KBRI) equipped with a TIRF module (Nikon H-TIRF), a high NA objective lens (Nikon Apo TIRF 100× NA 1.49 Oil) and an EMCCD camera (Andor iXon DU-897). Lasers with wavelengths of 488 nm were used to excite GFP. An additional barrier filter was installed in front of EMCCD to improve the signal-to-noise ratio in the sample's background autofluorescence. The exposure and EM gain of EMCCD were set to 100 ms and 300, respectively, to obtain clear images that could distinguish single-molecule emission signals from the background signal. The laser power was maintained at approximately 5 mW after the objective lens. For single-molecule photobleaching event analysis, time-series TIRFM images were acquired at 7 Hz from the GFP channel.

A custom-built image analysis software was used to identify protein complexes, find the central locations, calculate fluorescence intensity traces and count photobleaching events. The identification and centring were performed in the initial frame. Complexes that are well separated from the surrounding complexes and have well-distinguishable bleaching events were selected. Intensity trace was calculated as the pixel sum in a ROI with 5 × 5 pixels in each frame after applying a circular digital mask corresponding to 800 nm. To count photobleaching events, the intensity traces were convoluted with the first derivative-of-Gaussian wavelets, and then positions of the deep valleys were counted. Simultaneous photobleaching event of multiple fluorophores was compensated for by considering the step height calculated from the piecewise constant between two nearby events.

The distribution of photobleaching events was fitted with the discrete probability distribution model of binomial distribution with a specific number of total subunits. The likelihood of observing *k* bleaching steps, if a total of *n* steps is possible, can be expressed as a binomial distribution,

4.1 $$P(k)=P(k|n,\;p)=\frac{n!}{(n-k)!k!}\;p^{k}q^{n-k},$$

where *P*(*k*) represents the binomial distribution probability as a function of *k*, *n* is the number of total subunits expected, *p* represents the probability of detecting an existing subunit and *q* = (1 − *p*) represents the probability of not detecting an existing subunit. And the goodness of the fit can be evaluated by the SS for each model,

4.2 $$SS=\underset{k}{\sum }{\left[P(k)-P_{\mathrm{model}}(k)\right]}^{2},$$

where *P*(*k*) represents the fractional occurrence of measured events with *k* steps. And *P*_model_ (*k*) represents the value from the binomial fitting model.

## Data accessibility

This article has no additional data.
